# Supplementary material for: HIVprotI: an integrated web based platform for prediction and design of HIV proteins inhibitors
Source: J Cheminform. 2018 Mar 9;10:12. doi: 10.1186/s13321-018-0266-y (PMC5845081; doi:10.1186/s13321-018-0266-y)
Supplement: Supplementary file 2 — Additional file 2. Source code of HIVProtI web server. [file 13321_2018_266_MOESM2_ESM.zip › HIVprotI_Source-code/hivprotI/weka-3-6-6/doc/weka/core/package-frame.html]

weka.core


weka.core

Interfaces 
  
*AdditionalMeasureProducer*
  
*CapabilitiesHandler*
  
*Copyable*
  
*DistanceFunction*
  
*Drawable*
  
*EnvironmentHandler*
  
*JythonObject*
  
*JythonSerializableObject*
  
*Matchable*
  
*MultiInstanceCapabilitiesHandler*
  
*OptionHandler*
  
*Randomizable*
  
*RevisionHandler*
  
*Summarizable*
  
*TechnicalInformationHandler*
  
*Undoable*
  
*WeightedInstancesHandler*

Classes 
  
AbstractStringDistanceFunction
  
AlgVector
  
AllJavadoc
  
Attribute
  
AttributeExpression
  
AttributeLocator
  
AttributeStats
  
BinarySparseInstance
  
Capabilities
  
ChebyshevDistance
  
Check
  
CheckGOE
  
CheckOptionHandler
  
CheckScheme
  
CheckScheme.PostProcessor
  
ClassDiscovery
  
ClassDiscovery.StringCompare
  
ClassloaderUtil
  
ContingencyTables
  
Copyright
  
Debug
  
Debug.Clock
  
Debug.DBO
  
Debug.Log
  
Debug.Random
  
Debug.SimpleLog
  
Debug.Timestamp
  
EditDistance
  
Environment
  
EuclideanDistance
  
FastVector
  
FindWithCapabilities
  
GlobalInfoJavadoc
  
Instance
  
InstanceComparator
  
Instances
  
Javadoc
  
Jython
  
ListOptions
  
ManhattanDistance
  
MathematicalExpression
  
Matrix
  
Memory
  
NormalizableDistance
  
Optimization
  
Option
  
OptionHandlerJavadoc
  
PropertyPath
  
PropertyPath.Path
  
PropertyPath.PathElement
  
ProtectedProperties
  
Queue
  
RandomVariates
  
Range
  
RelationalLocator
  
RevisionUtils
  
SelectedTag
  
SerializationHelper
  
SerializedObject
  
SingleIndex
  
SparseInstance
  
SpecialFunctions
  
Statistics
  
Stopwords
  
StringLocator
  
SystemInfo
  
Tag
  
TechnicalInformation
  
TechnicalInformationHandlerJavadoc
  
Tee
  
TestInstances
  
Trie
  
Trie.TrieIterator
  
Trie.TrieNode
  
Utils
  
Version

Enums 
  
Capabilities.Capability
  
RevisionUtils.Type
  
TechnicalInformation.Field
  
TechnicalInformation.Type

Exceptions 
  
NoSupportForMissingValuesException
  
UnassignedClassException
  
UnassignedDatasetException
  
UnsupportedAttributeTypeException
  
UnsupportedClassTypeException
  
WekaException
